# Translation, adaptation, and psychometric evaluation of the Quality of Life in a Child’s Chronic Disease Questionnaire for the Swedish context

**DOI:** 10.1007/s00431-024-05888-7

**Published:** 2024-12-12

**Authors:** Karin Blomberg, Małgorzata Farnik, Mats Eriksson

**Affiliations:** 1 Faculty of Medicine and Health, School of Health Sciences, Örebro University, S-70182 Örebro, Sweden; 2 Medical University of Silesia, Katowice, Poland

**Keywords:** Psychometric evaluation, Translation, Questionnaire, Quality of life, Family, Chronic disease

## Abstract

The aim of this study was to translate, adapt, and psychometrically evaluate the Quality of Life in a Child’s Chronic Disease Questionnaire (QLCCDQ) for the Swedish context. The QLCCDQ was translated into Swedish and adapted to the Swedish context. Data for psychometric testing were obtained through a survey of parents of children and adolescents (*n* = 627) with chronic diseases: asthma and type 1 diabetes mellitus, with a total of 173 responses (27.6%). Face and content validity of the instrument was assessed, and floor and ceiling effects were measured. Internal consistency was measured with Cronbach’s alpha and an exploratory factor analysis (EFA) was conducted. The EFA gave a two-factor solution with an eigenvalue > 1 explaining 73.9% of total variance for the Swedish version. The new subscales are family life and activities (eight questions) and emotions and symptoms (four questions). Three questions concerning anxiety, worry, and guilt loaded < 0.6 and were excluded.

*Conclusion*: The study concludes that the Swedish version of the QLCCDQ is a reliable and valid questionnaire. The instrument may be useful for clinical screening of families who have the greatest need for supportive interventions. However, this should be further evaluated.

**Table:** 

**What is known:** *• A child's chronic disease influences quality of life of its family members.* *• Few instruments are designed to measure the impact on families.* **What is new:** *• The Swedish version of the Quality of Life in a Child’s Chronic Disease Questionnaire has two subscales compared to the original's five.* *• The instrument may potentially be useful for clinical screening of families who have the greatest need for supportive interventions.*

**What is known:**

*• A child's chronic disease influences quality of life of its family members.*

*• Few instruments are designed to measure the impact on families.*

**What is new:**

*• The Swedish version of the Quality of Life in a Child’s Chronic Disease Questionnaire has two subscales compared to the original's five.*

*• The instrument may potentially be useful for clinical screening of families who have the greatest need for supportive interventions.*

## Background

It is well known that an illness of a child influences health, wellbeing, and quality of life (QoL), i.e., overall life satisfaction [1] of family members [2, 3]. This includes emotional and financial aspects, family relations, education, work, and leisure and social activities [2–5]. A reciprocal impact has also been shown to exist between family relationships and management of illness [6–8]. Hence, protective factors such as family closeness and supportive family relations are considered factors that support good disease management, while intrafamilial conflicts and external stress are factors that may impair the management of the disease [9–11].

Therefore, when a child becomes ill with a chronic disease, the whole family is affected, in particular the child’s parents, who play an important role in establishing and maintaining cooperation between the child and health care professionals, and also in coping with the disease in everyday life. Parents have described an increased level of distress and limitations in daily activities with regard to their children’s chronic disease [12, 13]. However, this impact on families is often unrecognized and underestimated, as reported by Golics et al. [4] in a review of 158 studies. Several nursing interventions that focus on the family have been developed in different contexts of illness, including chronic child diseases [14]. The interventions are largely a series of supportive sessions, but they could also be web-based or sent as a therapeutic letter to the families [15]. This highlights the importance of evaluating effects on families and psychosocial aspects of parental functioning.

Health-Related Quality of Life (HRQoL) is a multidimensional concept including social, emotional, and physical functioning or well-being, related to the patient’s health state [16]. Several instrument have been developed to assess HRQoL in children [17]. Examples are Disabkids [18] or PedsQL [19], which both exist in a version that the child can answer, and a parallel proxy version for the parents. These instruments focus on the HRQoL of the child, but a few previous instruments aim to measure how the HRQoL of the family is affected by their child’s illness. PedsQoL [20] has a module for measuring the impact of chronic illness on parents’ quality of life and family functioning, also available in Swedish language [21], and more recently, the Family Reported Outcome Measure (FROM-16) [22]. Researchers has also used general QoL-instruments like the World Health Organization Quality of Life Questionnaire (WHOQOL) to investigate parents’ quality of life when having a child with a chronic disease [23]. To fill this gap, the Quality of Life in a Child’s Chronic Disease Questionnaire (QLCCDQ) has been developed to help understand the impact of chronic child diseases on the families’ daily life and wellbeing [24]. The QLCCDQ deals with problems and limitations in daily life that concern parents of children with chronic disease. The instrument is a self-reported measure of parents’ HRQoL and consists of 15 questions covering five subscales: emotions (four questions), symptoms (three questions), family roles (two questions), social roles (three questions), and occupational roles (three questions). The last three subscales are also combined into role limitations (eight questions). The QLCCDQ is scored on a 7-point Likert scale from 1 (most bothered or limited) to 7 (not bothered or limited), with lower values indicating lower HRQoL. The instrument has shown good internal consistency, test–retest reliability, and construct validity [24]. It has been utilized in studies on pediatric asthma [25], alopecia [26], stammering [27], and vitiligo [28].

Two major chronic diseases in children are asthma and diabetes. In Sweden, approximately 7% of all 10-year-old children are diagnosed with asthma, compared to 9% in Europe [29] and 0.1% of adolescents have diabetes compared to 0.3% in Europe [30]. Both conditions utilize considerable health care resources and affect the life of the families. We undertook to adapt the QLCCDQ for use by Swedish parents of children and adolescents with chronic diseases and to make it available for research and planning of family interventions. The aim of this study was therefore to translate, adapt, and psychometrically evaluate the QLCCDQ for the Swedish context.

## Method

### Design

This research is part of a project assessing the potential impact of chronic disease in children on the life situation and QoL of these children and their families [31]. Data for the psychometric evaluation study were collected using the QLCCDQ translated into Swedish and adapted for use in the Swedish context. In this first step, an explanatory factor analysis (EFA) was used to psychometrically evaluate the QLCCDCQ in a new context (Swedish population). EFAs aim to determine factor loadings and the latent underlying relationships between the items, especially when the structure lacks a strong theoretical foundation [32]. In this study, the sample consisted of two groups of common chronic diseases in children even if the objective here was not to testing differences among parents of children with diabetes and asthma.

### Phase 1: translation and adaptation of the QLCCDQ for the Swedish context

The original, English-language QLCCDQ instrument was received from the developer (MF) who gave permission to translate it into Swedish and use it in Sweden. The translation and cultural adaptation followed guidelines from the International Society for Pharmacoecomics and Outcomes Research (ISPOR) [33]. The first translation from English into Swedish was performed by two of the researchers (KB and ME) who speak Swedish as their native language and are proficient in English. A forward translation was done by a native English-speaking, Swedish researcher, the aim being to identify any discrepancies and seek agreement between the translations. This process resulted in a preliminary version of the Swedish instrument.

### Phase 2: face and content validity

The first Swedish version of the QLCCDQ was then evaluated for face and content validity in a sample of parents of children and adolescents with asthma and diabetes mellitus using cognitive interviews [34, 35]. This phase took place at the Department of Paediatrics at a university hospital in mid-Sweden. Eligible persons were identified by a nurse working at the Department and recruited by purposive sampling [36]. The exclusion criterion was inability to understand Swedish. The first seven parents who expressed an interest in attending an interview received information about the study, and a time for the interview was arranged. All seven parents gave informed consent (both orally and in writing) to participate in an interview. The sample size was considered sufficient with regard to ISPOR guidelines for cognitive debriefing interviews in translation and validation of measurements [33].

The parents filled in the questionnaire and each item was discussed with the researcher. While answering the questionnaire, parents were asked to “think aloud” [34, 35], i.e., to express their thoughts and whether they hesitated at any question or perceived any question as unclear. The participants were also observed during the interview. Probing questions were asked, for example: “You seem to hesitate; do you feel this question is difficult to answer?” (If the response was in the affirmative, the researcher asked, “In what way?”) All interviews lasted between 12 and 15 min, were conducted in a separate room at the hospital, and were audio-recorded. In the analysis of the interviews, all recordings were listened to, and notes were taken regarding parents’ reflections and comments on the instrument. The focus in the analysis was on whether each of the questions was clear and appropriate and whether the instrument seemed difficult for parents to understand and complete.

### Phase 3: psychometric evaluation

A sample of parents of children and adolescents with chronic diseases, namely, asthma (*n* = 389) and type 1 diabetes mellitus (*n* = 238), was recruited from the Department of Paediatrics at a university hospital in mid-Sweden. The inclusion criterion was having a child under 19 years of age with one of these diseases who was on the patient list of the hospital. The parents were sent a letter with an information sheet about the study in September 2016 (asthma group) and between October 2018 and May 2019 (diabetes group). The letter contained an information sheet about the study, as well as the Swedish version of the QLCCDQ and demographic questions such as age, gender, year of diagnosis, together with a prepaid response envelope. A reminder was sent to all eligible respondents 2–4 months after the first letter. The final response rate was 27.6%.

### Statistical analyses and psychometric testing

Data are presented as mean, median, range, number, and percentage, depending on data type and distribution. Student’s *t*-test or Mann–Whitney *U*-test were used to test statistical significance. Cronbach’s alpha was calculated, and an exploratory factor analysis (EFA) was conducted for each subscale and for the total instrument [37]. An EFA with oblimin rotation and Kaiser normalization was conducted to determine the factor loadings and the underlying relationships between the QLCCDQ items. A principal component analysis was used for factor extraction. The sample size was in accordance with recommendations (rules of thumb vary, from four to ten subjects per variable, with a minimum of 100 subjects to ensure stability of the variance–covariance matrix). Therefore, the number of respondents was adequate in relation to the number of items. All statistical calculations were conducted using SPSS version 28 (IBM Corp., Armonk, NY, USA).

### Internal consistency

Internal consistency is the degree of interrelatedness among the items in an instrument and was measured using Cronbach’s alpha. Cronbach’s alpha was calculated for the subscales and for the whole instrument. A Cronbach’s alpha between 0.70 and 0.95 is considered to be good [38].

### Floor and ceiling effects

Floor and/or ceiling effects occur if more than 15% of respondents achieve the lowest or the highest possible score [38]. Floor and ceiling effects were calculated for the whole instrument and for each subscale in the QLCCDQ.

### Ethical considerations

The study was performed in accordance with the Declaration of Helsinki and after approval from the Regional Ethical Review Board in Uppsala (reg. no. 2013/505). Participants in the cognitive interviews were given both verbal and written information about the study and were asked for written informed consent before the interviews. Participants in the survey were informed about the study in writing and indicated their informed consent by answering and returning the questionnaire.

## Results

### Translation

The 15 questions in the QLCCDQ were translated into Swedish by two independent translators and the two versions were discussed and merged into one version by the research group. This version, which was considered to be understandable to a Swedish readership, was then back-translated into English and checked against the original instrument. No changes were deemed necessary in this process.

### Face and content validity

Seven parents (two men, five women, mean age 42.2 years) of children with diabetes participated in the cognitive interviews. They perceived the majority of the questions as relevant and clear to understand. Two parents commented that the instruction of the instrument was somewhat unclear, as it was not clear whether the ratings should be based on how the situation was now or in the last 2 weeks. One parent commented that the answers might depend on the child’s trajectory of illness. For instance, they might differ depending on whether the questionnaire was being answered at the beginning of the child’s disease or when the child had the disease for several years. Another parent commented that question 6 about the experience for siblings was unclear because the entire family was affected. None of the parents made suggestions for how to strengthen the readability of questions. Altogether, face and content validity were established as the parents perceived the items in the QLCCDQ to be relevant to their life situation.

### Study participants

The questionnaire was answered by one parent each in 174 families in which at least one child or adolescent had a chronic disease: asthma (117 families, 67%) or type 1 diabetes mellitus (57 families, 33%) (see Table 1). The mean age of the children was 11.6 years, standard deviation (SD) 3.1.

**Table 1:** Demographics of responding families

	Diabetes (*n* = 57)	Asthma (*n* = 117)	*p*-value
Child’s age group, *n* (%)			** < 0.001**
* 0*–*7 years*	7 (12)	0 (0)^a^	
* 8*–*13 years*	27 (47)	88 (75)^a^	
* 14*–*18 years*	24 (41)	29 (25)	
Child’s gender, *n* (%)			0.096
* Male*	31 (53)	71 (65)	
* Female*	27 (47)	38 (35)	
Who responded?, *n* (%)			0.119
* Father*	14 (26)	19 (17)	
* Mother*	39 (74)	91 (79)	
* Other*		5 (4)	

^a^Statistically significant differences regarding demographics were found within these groups (with Bonferroni correction)

Significance in bold

### Exploratory factor analysis

**Fig. 1:**
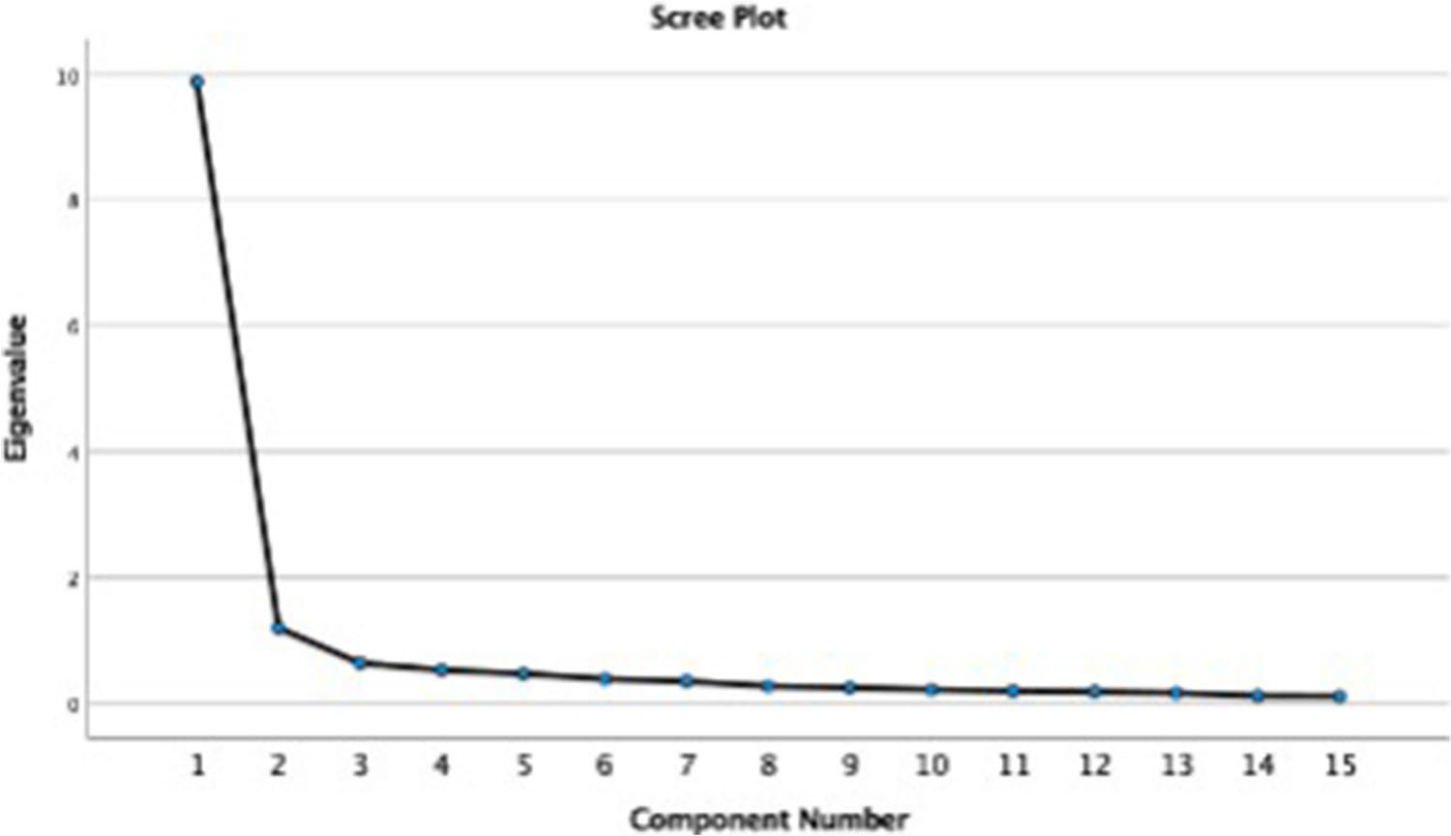
Scree plot supporting a two-factor solution

The EFA showed a Kaiser–Meyer–Olkin measure of sampling adequacy of 0.944 with Bartlett’s test of sphericity of < 0.001. Both the slope of a scree plot (Fig. 1) and the EFA with oblimin rotation and Kaiser normalization indicated that a two-factor solution best suited the Swedish version of the QLCCDQ with an eigenvalue > 1, explaining 73.9% of the variance. Questions 7, 9, and 11 loaded < 0.6 in both factors and could therefore be excluded (Table 2). Questions 7 and 9 concerned anxiety and worry about the child and question 11 concerned personal guilt in parents and hence was quite different from the rest of the questions. It was therefore decided that these questions should be omitted from the Swedish version.

**Table 2:** Factor loadings using oblimin rotation. Questions listed here are from the original, English-language questionnaire. Bold figures indicate the suggested domain. Questions 7, 9, and 11 loaded < 0.6 in both factors and have therefore been excluded from the new Swedish-language version

	Factor	1	2
How often in the past 2 weeks …			
1	Have you given up meeting your friends because of your child’s disease?	**0.839**	− 0.024
2	Have you refrained from hobbies/entertainment because of your child’s disease?	**0.779**	0.121
3	Have you been depressed because of your child’s disease?	0.364	**0.603**
4	Did the child show any worrying symptoms?	− 0.049	**0.872**
5	Have you noticed that symptoms affect your child’s physical activity?	− 0.029	**0.801**
How often, in general …			
6	Do you struggle to find time to spend with other family members (spouse, another child) because of your child’s disease?	**0.680**	0.252
7	Have you experienced anxiety due to your child’s disease?	0.412	0.542
8	Have you found your child suffering due to their disease?	− 0.026	**0.927**
9	Have you been worried or concerned about your child’s future, due to their disease?	0.382	0.587
10	Does your child’s disease have an impact on your career?	**0.800**	0.095
11	Do you feel guilty due to your child’s disease?	0.476	0.413
12	Does your child’s disease have an impact on your family’s financial situation?	**0.718**	0.207
Please indicate how much you have been limited by your child’s disease in listed activities in the past 2 weeks			
13	Household activities (housework, shopping, cleaning)	**0.912**	− 0.124
14	Social activities (going to church, cinema, visiting friends)	**0.973**	0.133
15	Work-related activities	**0.874**	0.024

### New Swedish scale

As a result of the factor analysis, a Swedish version of the QLCCDQ-scale (S-QLCCDQ) was constructed, with 12 questions forming two subscales: family life and activities (eight questions) and emotions and symptoms (four questions) (Table 3).

**Table 3:** Items in the original and Swedish language version of QLCCDQ

		Original scale						Swedish scale	
		Emotions	Symptoms	Family roles	Social roles	Occupational roles	Role limitations	Family life and activities	Emotions and symptoms
How often in the past 2 weeks …									
1	Have you given up meeting your friends because of your child’s disease?				x		x	x	
2	Have you refrained from hobbies/entertainment because of your child’s disease?				x		x	x	
3	Have you been depressed because of your child’s disease?	x							**x**
4	Did the child show any worrying symptoms?		x						**x**
5	Have you noticed that symptoms affect your child’s physical activity?		x						**x**
How often, in general …									
6	Do you struggle to find time to spend with other family members (spouse, another child) because of your child’s disease?			x			x	x	
7	Have you experienced anxiety due to your child’s disease?	x							
8	Have you found your child suffering due to their disease?		x						**x**
9	Have you been worried or concerned about your child’s future, due to their disease?	x							
10	Does your child’s disease have an impact on your career?					x	x	x	
11	Do you feel guilty due to your child’s disease?	x							
12	Does your child’s disease have an impact on your family’s financial situation?					x	x	x	
Please indicate how much you have been limited by your child’s disease in listed activities in the past 2 weeks									
13	Household activities (housework, shopping, cleaning)			x			x	x	
14	Social activities (going to church, cinema, visiting friends)				x		x	x	
15	Work-related activities					x	x	x	

### Internal consistency

The internal consistency of the two subscales and the total S-QLCCDQ varied from 0.89 to 0.95 (Table 4).

**Table 4:** Cronbach’s alpha for the subscales and the total Swedish QLCCDQ. Question numbers refer to the original QLCCDQ in Table 2

Domain	Questions	Cronbach’s alpha
Family life and activities	1, 2, 6, 10, 12, 13, 14, 15	0.945
Emotions and symptoms	3, 4, 5, 8	0.890
Total S-QLCCDQ	1, 2, 3, 4, 5, 6, 8, 10, 12, 13, 14, 15	0.949

### Floor and ceiling effects

Both subscales, as well as the total S-QLCCDQ score, were positively skewed and had a substantial ceiling effect. For family life and activity, 66.9% of the respondents reported a score > 6, the corresponding numbers for emotions and symptoms was 43.6%, and for the total QLCCDQ, 60.5%.

## Discussion

This is the first study, as far as we know, to translate the QLCCDQ into and adapt it for another language and culture. The Swedish version of the QLCCDQ has now been validated, with positive psychometric results, and the instrument is available for use. The process of translation and adaptation has enhanced the clarity and consistency of the instrument for Swedish users. The pre-testing, including the cognitive interviews, demonstrated both face and content validity as the parents deemed the majority of the questions clear and appropriate and, overall, the instrument made sense to the parents. However, the instruction of the instrument needs to be clarified as it was somewhat unclear, whether the ratings should be based on how the situation was now or in the last 2 weeks.

The psychometric evaluation shows that the Swedish version of the QLCCDQ is a reliable and valid questionnaire, based on a two-factor solution. The internal consistency of the different subscales was closely related to those in the English language validation study; however, no factor analysis was performed for the original dataset [11].

However, it is suggested that three questions (7, 9, and 11) should be omitted from the Swedish version as it loaded < 0.6 in both factors. Our interpretation is that the lower reliability of question 11 mainly has conceptual reasons. The question concerns personal guilt in parents and, therefore, is quite different from the other questions. The low loading of the item could be explained by differences between cultures as guilt can be a sensitive subject and can have different meanings among people of different cultures. However, none of the parents commented on this question in the cognitive interviews. Questions 7 and 9 both concern personal feelings: worry and anxiety about the child’s disease, which might explain why they loaded low in this scale focusing on impact on the family quality of life.

When comparing with the subscales in the original QLCCDQ we find that the questions in social roles, family roles, and occupational roles all go together in family life, while the questions in emotions and symptoms are gathered in the S-QLCCDQ subscale with the same name: emotions and symptoms. Reducing five subscales into two is a result of the factor analysis but still covers the important aspects in quality of life for families [39]. The difference between the original and Swedish versions might be explained by the lack of a theoretical foundation in the original structure.

In general, the instrument showed acceptable reliability despite relatively few items (*n* = 12). Shorter scales are at risk of low reliability because the mean of the random errors associated with each item will be closer to zero, the more items there are in the scale [40]. The findings indicate that the Swedish version of the QLCCDQ is clear and easy to understand and this increases the instrument’s usability in clinical research. Another strength may be that the instrument covers different domains, both emotional and social aspects of family life, within the same tool, which enhances the possibility to compare the effectiveness of different interventions [41]. Another strength of the instrument is that it seems to work regardless diagnosis, i.e., asthma or diabetes.

The instrument has potential for clinical use. As the parents perceived it as a relatively easy and quick-to-complete instrument, it may have clinical relevance. We know that many families face several challenges when a child is affected by a lifelong chronic disease such as asthma or diabetes [4, 13]. It is well known that support is necessary for daily living and for coping with the disease [4], to achieve the best possible QoL for all family members. To enable support to families with the greatest needs it may be helpful to use the S-QLCCDQ as a screening instrument. However, this needs to be further evaluated. Further psychometric studies are also needed, to evaluate the instrument’s stability over time and distinguish between true change and change due to measurement error in the S-QLCCDQ. Future studies could also compare S-QLCCDQ with the Swedish version of PedsQoL [21]. Another tentative future task could be to develop an instrument where the children assess the quality-of-life in the family. A next step in psychometric testing is also to perform a confirmatory factor analysis (CFA), as well as hypothesis testing, to evaluate and further develop the instrument.

One strength of this study is the sample size (*n* = 174) in two groups of common chronic diseases among children. This is slightly over the recommendation of ten respondents per item in psychometric testing in order to reach a stable co-variation among the items [40]. Additionally, including two groups of common chronic diseases in the sample strengthen the results that the instrument seems to work regardless diagnosis. A limitation of this study is the low response rate, and it can be disputed whether a web-based survey would have increased the response rate.

## Conclusions

The translation, adaptation, and psychometric testing of the QLCCDQ for the Swedish context has shown an acceptable construct validity in the instrument. However, the analysis shows that three questions should be omitted from the final Swedish version of the QLCCDQ. The results also show that the instrument may potentially be useful for clinical screening of families who have the greatest need for supportive interventions. However, this should be further evaluated. Further psychometric studies are also needed to evaluate the instrument’s stability over time.

## Data Availability

Data from the cognitive interviews can not be shared, for ethical reasons. Data for the psychometric analysis can be shared upon reasonable request to the corresponding author.
